# Knowledge, Attitude, and Practice about Emergency Contraception among Health Staff in Bushehr State, South of Iran

**DOI:** 10.5539/gjhs.v6n1p52

**Published:** 2013-10-12

**Authors:** Fatemeh Najafi-Sharjabad, Abdollah Hajivandi, Mohammad Rayani

**Affiliations:** 1 Faculty of Health, Bushehr University of Medical Sciences, Bushehr, Iran; 2 Department of Biostatistics, School of Medicine, Bushehr University of Medical Sciences, Bushehr, Iran

**Keywords:** knowledge, attitude, practice, emergency contraception, Bushehr, Iran

## Abstract

Emergency Contraception (EC) is used within a few days of unprotected sex to prevent an unintended pregnancy. About one quarter of pregnancies in south of Iran are unintended. EC is important option that women can use after unprotected sex or contraceptive failure for preventing of unplanned pregnancies and adverse maternal and perinatal health outcomes. Health staff have influence on women’s contraceptive behavior and their knowledge and attitudes about EC can affect women’s contraceptive behaviors. Data are lacking about the knowledge, attitude and practice of hormonal EC method among health staff in Bushehr state, south of Iran. A cross-sectional study using self administered questionnaire was conducted. A sample of 170 health staff were surveyed. The mean age of respondents was 30.6±5.1. Overall 6.5% of participants had poor knowledge, 25.2% moderate knowledge, 68.3% good knowledge about EC. Half of participants had positive and half had negative attitude towards the EC method. Midwives and family health workers were more knowledgeable (p<0.05) and more frequently counseled women about EC than general practitioners (GPs) (p<0.001). The most cited reason for EC prescriptions were rupture condom and none use of contraception. Our findings showed despite of majority of health staff had good knowledge about EC, their knowledge about the indications for prescription of EC and its side effects was inadequate. The educational efforts for health staff should be focused more on the specific aspects of EC method. GPs also should be more involved in family planning program.

## 1. Introduction

Emergency Contraception (EC) is a back-up method of contraceptive which women can use within the first few days after unprotected intercourse to prevent an unwanted pregnancy. EC methods include hormonal and mechanical method. Hormonal EC pills contain higher levels of a hormone found in daily oral hormonal contraceptives (Brunton & Beal, 2006). Two common methods of hormonal EC include the Yuzpe regimen and plan B. Yuzpe regimen consists of the administration of two doses of combined oral contraceptive pills (each dose containing 100 μg of ethinyl estradiol and 1mg norgestrel) taken 12 hours apart but within 72 hours of the unprotected sex (Yuzpe & Lancee, 1977). Plan B is recommended by World Health Organization. It consists of 1.5 mg levonorgestrel as a single dose alone (World Health Organization, 2005). These pills also can be used as EC pills: a special EC product with the progestin levonorgestrel, EC product with estrogen and levonorgestrel, and Combined Oral Contraceptives (OCPs) (WHO, 2007).

EC pills sometimes are referred to as “morning-after” or “postcoital” pills and should be initiated as soon as possible after intercourse because the efficacy declines substantially with time (International Consortium for Emergency, 2004). EC pills are 75% - 95% effective if taken within 72 hours of unprotected intercourse (Trussell et al., 1999). EC is important option which can prevent physical and psychological consequences of unwanted pregnancy (Westley et al., 2007). It is difficult for many women to obtain EC pill within the recommended time frame. Advance provision could bypass some obstacles to timely use EC pill without delay (Polis, et al., 2007).

The need for EC may arise when no contraceptive method has been used, contraceptive failure or incorrect use, such as incorrect use of condom or breakage, three or more consecutive missed combined oral contraceptive pills (OCP), delay more than two weeks for injectable method, failed withdrawal method, intra uterine device (IUD) expulsion and case of sexual assault when woman is not protected by contraception (World Health Organization, 2005).

Each year 42 million abortions are estimated to take place, 22 million safely and 20 million unsafely. Unsafe abortion accounts for 70,000 maternal deaths each year and five million women are hospitalized each year for treatment of abortion complications such as hemorrhage and sepsis (Shah & Ahman, 2009). Maternal mortality ratios due to unsafe abortion are higher in regions with restricted abortion laws than in regions with no or few restrictions on access to safe and legal abortion (Shah & Ahman, 2009; Erfani & McQuillan, 2008).

Despite of dramatically decline in fertility in recent decades in Iran, but limited access to legal abortion leads many women with unintended pregnancies to resort clandestine, unsafe abortions. According to a recent analysis of nationally representative data abortion has been estimated 73,000 annually among married women aged 15-49 in Iran (Erfani & McQuillan, 2008).

A study conducted in Bushehr state in south of Iran revealed that 24% of pregnant women reported unwanted pregnancy. The most frequently contraceptive methods used prior pregnancy were withdrawal method (41%), Oral Contraceptive Pill (OCP) (21.3%), and condom (13.8%) (Norouzi, 2005). Proper use of EC may be an effective way to reduce the number of unwanted pregnancies and induced abortions (Haspels, 1994). It is critical for women to have awareness about timely access to EC pills preferably before they need to use them (Virjo et al., 1999). EC also can be served as a bridge into the health care system and a way to obtain a regular contraceptive method for women who do not use one (Grossman & Grossman, 1994).

The World Health Organization recommends that reproductive health services offer EC as part of their routine services (Van Look & von Hertzen, 1993). Health staff interact with large numbers of women and are reliable sources of information. They have influence on women’s contraceptive behavior and their knowledge and attitudes about EC can impede or promote women’s use of EC (Sills et al., 2000; Simonds & Ellertson, 2004; Kelly et al., 2008).

In Iran family health workers and midwives work in government health centers give information about contraceptive methods and offered contraception free of charge or for minimal payment. In Iran’s health care system a higher dose of combined oral contraceptive (Yuzpe method) is offered as EC method. Two common combined oral contraceptive pills that widely are used in Iran are low dose (LD) combined OCP including 0.3 mg levonorgestre and 30 µg ethinyl estradiol and high dose (HD) combined OCP including 0.5mg levonorgestrel and 50 µg ethinyl estradiol. There is a lack of published data about health staff’s knowledge of, attitude towards and provision of EC pills in the south of Iran, where unplanned pregnancies are persistent public health problems. The aim of this study was to assess the knowledge, attitude and practice of EC among health staff in Bushehr state in south of Iran.

## 2. Method

### 2.1 Study Design and Participants

This study used a descriptive cross-sectional design to determine the knowledge, attitude, and practice about EC method (Yuzpe Regimen) among health staff in Bushehr, South of Iran.

All health staff including general practitioners (GPs), midwives, family health workers who work in urban health centers were invited to participate in this study.

Based on Yamane’s sample size formula the required sample for this study was 160, where n is the sample size, N is the population size, and e is the level of precision. A 95% confidence level and P=0.5 are assumed for the equation, P is distribution of attributes in the population (Yamane, 1967).


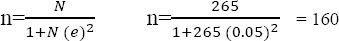


Out of nine cities in Bushehr state six cities were selected. A simple random sampling by lottery method was used for selection of cities. There were 23 urban health centers in six selected cities. The study took place over eight week period in April and May 2010 among health staff at 23 urban health centers in Bushehr state in south of Iran. Stratified random sampling was used for selection of subjects. Prior to recruitment of the subjects, the head of family health department in Bushehr state was informed about the objective of the study. Briefing session was held at the work place and the objectives of study and the eligibility recruitment to participate in the study were explained to the all health staff. A coordinator also was selected from each health office in selected city to facilitate data collection procedure.

### 2.2 Measurement

The questionnaire was adapted from a World Health Organization guideline for EC and previously published studies (WHO, 2005; Sevil et al., 2006; Ebuehi et al., 2006). The questionnaire included 28 questions on various aspects of EC method.

There were five items on demographic characteristic of health staff, ten items on knowledge of EC, eight items on attitude towards EC, and five items on EC practice. Practices of EC were a series of questions about the advance provision, prescription and counseling of EC method, screening women for unprotected sex, attendance on family planning training program.

Content validity was checked with two experts in public health working in Bushehr state health department. The experts checked whether each item on a scale is congruent with (or relevant to) the construct, computing the percentage of items deemed to be relevant for each expert, and then taking an average of the percentages across experts. This has been referred to as the average congruency percentage (ACP) (Popham, 1978). The ACP of 90% or higher would be considered acceptable (Waltz et al., 2005). The ACP for this study was 95%.

Pretesting of the questionnaire was carried out among 15 midwives, and five GPs working in Bushehr health center to ensure the respondents understood the items and to measure the reliability (internal consistency) of the scales in the questionnaire.

For pretesting the Cronbach’s alpha coefficient was 0.74 for knowledge on EC, and 0.71 for attitude scale. The Cronbach’s alpha value from the sample of 170 was 0.78 for knowledge on EC, and 0.72 for attitude towards EC. The Cronbach’s alpha value for both scales is above 0.70 which indicated adequate reliability of the questionnaire (De-Vellis, 2003).

For evaluation of EC knowledge the respondents were requested to respond on the individual statements using “Yes” or “No” or “Do not know”. Knowledge on EC includes awareness of EC, dosage, time frame, indication of EC usage, side effects and its mechanism of action.

Respondents were given one point for answering correctly and no points for answering wrongly or that they did not know. Respondents’ knowledge scores on EC were summed and then categorized as good (above the 75^th^ percentile), moderate (50^th^ to 75^th^ percentile) or poor (below 50^th^ percentile). Attitude scores were measured by composite score of 8 items using the five point Likert scale from one for strongly disagree to five for fully agree. Attitude scores above mean were categorized as positive and scores below mean were categorized as negative attitude.

In this study attitude of EC was defined health staff’s opinion about various aspects of EC usage such as whether EC causes abortion, safety of EC, its effect on regular contraceptive practice, religious concern for EC prescription, training reproductive women about EC, and effect of using EC on increasing risky sexual behavior and decreasing condom usage.

### 2.3 Ethical Considerations

Ethics Committee of Bushehr University of Medical Sciences granted ethical approval for the study. The self administered questionnaires were distributed after obtaining verbal consent. The health staff were assured that the questionnaire is anonymous and confidential.

### 2.4 Data Analysis

Data were coded and analyzed using SPSS, IBM Statistics, version 19.

Descriptive analysis was presented in term of frequency, percentage, means, and standard deviations. Chi-square tests were used for the comparison of proportions and for examining the association between categorical variables. Associations and differences were considered statistically significant at p < 0.05.

## 3. Results

### 3.1 Response Rate

In order to maximize study participation and collecting data for this research head of family health department in Bushehr state sent request by mail to the respective health centers. From 173 invited health staff 170 accepted to participate in this study. All 170 health providers completed and returned the questionnaires, giving a total participation rate of 98%.

The mean age of respondents was 30.6 ± 5.1 (Mean ± SD) years and ranged from 21 years to 50 years. The average working years of the participants was 7.4 ± 2.3 (Mean ± SD) years (Table 1).

**Table 1:** Emergency contraception prescription by health staff according to socio demographic characteristics (n=170)

variable	Total(n=170)	EC pill prescription within last year		*χ^2^*	p-value
		**≤ 10 times** No. (%)	**>10times** No. (%)		
**Age (years)**					
20-29	76	28 (36.8)	48 (63.2)		
30-40	78	21 (26.9)	57 (73.1)	2.64	p>0.05
>40	16	7 (43.8)	9 (56.3)		
(Mean ± SD)	30.6± 5.1				
**Gender**					
Male	16	13 (81.3)	3 (18.7)		
Female	154	43 (27.9)	111 (72.1)	18.66	p<0.001
**Marital Status**					
Married	115	32 (27.8)	83 (72.2)		
Single	54	23 (42.6)	31 (57.4)	3.65	p> 0.05
**Job Category**					
GP	35	29 (82.9)	6 (17.1)		
Midwife	65	13 (20)	52 (80)	49.713	p<0.001
Family health worker	70	14 (20)	56 (80)		
**Working Experience**					
≤ 10 years	113	42 (37.2)	71 (62.8)		
>10 years	57	14 (24.1)	43 (75.9)	3.076	p>0.05
(Mean ± SD)	7.4 ± 2.3				

Significant level at p<0.05, GP= general practitioner

### 3.2 Emergency Contraception Practices

Table 1 shows the higher percentage of female health workers (72.1%) prescribed EC more frequently as compared to male health workers (18.7%; p<0.001).

The EC prescription differed by occupation. Midwives and family health workers prescribed EC more frequently as compared to GPs (p<0.001) (Table 1).

**Figure 1:**
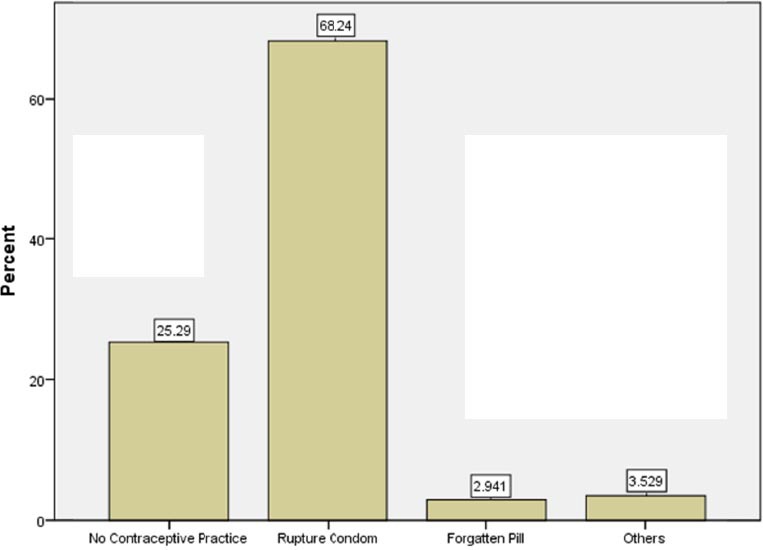
The most cited reason for emergency contraception prescription

**Figure 2:**
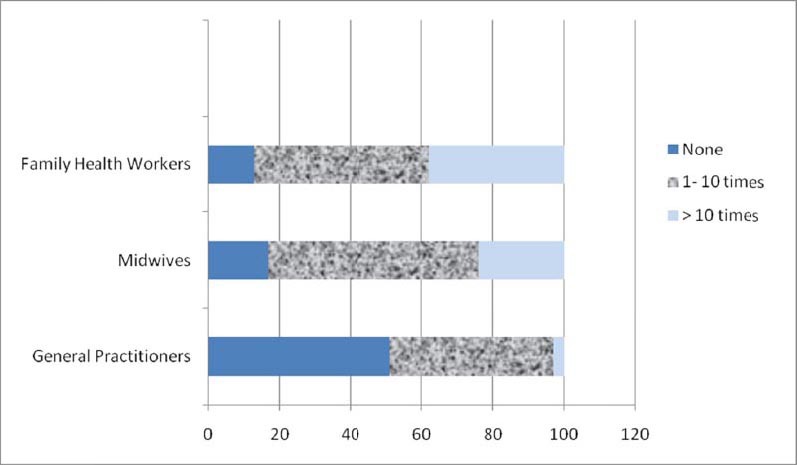
Health staff’s attendance on family planning training program

There were no significant difference in EC prescription by different age categories, marital status and working years. Midwives and family health workers (70%) more frequently reported advance provision of EC method compare to GPs (20%; p<0.001). Also, the higher percentage of midwives and family health workers (83%) counseled women about EC method often/always as compared to GPs (23%; p<0.001). Further, higher proportion of midwives and family health workers (48%) screened women for unprotected sex than the GPs (28.6%; p<0.05). The most common reported cases for EC prescription were rupture condom (68%) followed by no contraceptive use (25%), and forgotten pill (3%) (Figure 1). This study revealed GPs less frequently attended on family planning training program compare to nurses and midwives (Figure 2).

### 3.3 Knowledge of Emergency Contraception

Overall 6.5% had poor knowledge, 25.2% had moderate knowledge, 68.3% had good knowledge (Table 3). The level of knowledge differed by job categories, with a higher proportion of midwives and family health workers having good knowledge (71.1%) compared to GPs (57.1%; p<0.05) (Table 1).

**Table 2:** Distribution of correct response to knowledge questions on emergency contraception (Yuzpe Regimen)

Knowledge statement	GPs (n=35)	Midwives (n=65)	FH Workers (n=70)	Total (n=170)
	No. (%)	No. (%)	No. (%)	No. (%)
**Dosage**			
Four HD combined OCP (T)	35 (100)	62 (95.4)	68 (97.1)	165 (97)
Eight LD combined OCP (T)	34 (97.1)	63 (96.9)	67 (95.7)	164 (96.4)
**Timing of administration:**			
within 72 hours (T)	23 (65.7)	54 (83.1)	51 (72.9)	128 (75.3)
**Time interval:**			
The second dose of EC pill is taken after 12 hours (T)	32 (91.4)	63 (96.9)	67 (95.7)	162 (95.3)
**Indication:**			
Three or more consecutive OCP forgotten (T)	14 (40)	30 (46.2)	23 (32.9)	67 (39.4)
Rupture condom(T)	35 (100)	62 (95.4)	65 (92.9)	162 (95.3)
Delay injection more than 2 week (T)	11 (31.4)	33 (50.8)	32 (45.7)	76 (44.7)
**Efficacy:** 75% (T)	27 (77.1)	54 (83.1)	60 (85.7)	141 (82.9)
**Common side effect**: nausea (T)	34 (97.1)	62 (95.4)	64 (91.4)	160 (94.1)
**Mode of action:**				
EC prevent pregnancy via abortion(F)	27 (77.1)	59 (87.7)	53 (75.7)	137 (80.6)

T= true; F= false; HD, high dose pill including 0.5mg levonorgestrel and 50 µg ethinyl estradiol; LD, low dose pill including 0.3 mg levonorgestre and 30 µg ethinyl estradiol; GP= general practitioner; FH, family health; OCP, oral contraceptive pill

**Table 3:** Knowledge, attitude and practices of health staff about emergency contraception (n=170)

Variable	GP (n=35)	M/FHW(n=135)	*χ^2^*	**p-value**
	No. (%)	No. (%)		
**Knowledge of EC**				
Poor	4 (11.4)	7 (5.2)	3.125	p<0.05
Moderate	11 (31.4)	32 (23.7)		
Good	20 (57.1)	96 (71.1)		
**Attitude of EC**				
Negative	17 (48.6)	67 (49.6)
Positive	18 (51.4)	68 (50.4)	0.012	p>0.05
**Advance EC provision in past 12 month**				
≤ 10 times	28 (80)	41 (30.4)		
>10 times	7 (20)	94 (69.6)	28.39	p<0.001
**Counseling about EC**				
Never/Sometimes	27 (77.1)	23 (17)		
Often/Always	8 (22.9)	112 (83)	48.365	p<0.001
**Screening for unprotected sex**				
Never/Sometimes	25 (71.4)	70 (51.9)		
Often/Always	10 (28.6)	65 (48.1)	4.321	p<0.05

GP= general practitioner, M=midwife, FHW= family health worker

All GPs and majority of midwives and family health workers identified the dosage of EC pills. Sixty six percent of GPs, 83% of midwives and 73% of family health workers correctly answered that EC pills could be taken up to 72 hours after unprotected sex. The majority of all three occupations knew the time interval between of taking EC pills. When asked to identify appropriate candidates for using emergency contraception, all GPs, 93% of family health workers and 95% of midwives correctly answered EC pill could be used following condom breakage. But only 40% of GPs, 46% of midwives and 33% of family health workers correctly indicated the EC method would be appropriate after missing three or more consecutive regular contraceptive pills. We found that 31% of GPs, 51% of midwives and 45% of family health workers correctly answered that EC method could be used in cases with delay in injectable contraceptive methods. Further, a majority of all three occupations correctly knew that EC method (Yuzpe Regimen) is 75% effective in preventing pregnancy and nausea is common side effects of the method. In addition 77% of GPs, 88% of midwives and 76% of family health workers correctly rejected the statements that EC prevent pregnancy via abortion (Table 2).

### 3.4 Attitude towards Emergency Contraception

The mean attitude score was 28.44 ± 5.29. Attitude score of 84 (49.4%) of respondents was below mean (negative attitude) and 86 (50.6%) of respondents got score above mean (positive attitude). The minimum attitude score was 8 and the maximum of 40. A majority (84%) of disagreed that EC pill causes abortion. One third believed use of effective contraceptive method will decrease if people know about the method. One third of them thought use of EC pill encourage risky sexual behaviour and one quarter believed use of EC decrease the use of condom. Only a few cases (7%) had religious concern for prescription of EC pill. This study showed 77% of concerned repeated use of EC causes health risk. A majority (85%) of respondent had positive opinion that the reproductive women should be educated about EC methods. There was no significant difference on attitude toward EC between midwives and family health workers and GPs (p>0.05) (Table 3).

## 4. Discussion

This study was the first undertaken to elicit the knowledge, and attitudes of emergency contraception among health staff in Bushehr state, south of Iran. Overall high proportion of had good knowledge about EC method. Most of all three job categories knew the dosage of EC pill, time interval for taking EC pills, its effectiveness and mode of action of the method. However we found there is a lacking of adequate knowledge of some indication of EC pill among GPs, midwives and family health workers. Most of health staff did not have information that EC pill can be used after forgotten oral contraceptive pills or delayed injectable method.

Our study found half of health staff had positive attitude toward EC. In comparison to previous published studies in Iran the health providers were more knowledgeable and revealed more favorable attitude of EC. In a descriptive study among 216 health providers in Iran, only 10% of health providers had good knowledge of EC and majority had neutral attitude toward EC (Rahimikian, 2006).

Study by Jamali and Azimi (2005) among 150 GPs and midwives in family planning clinics in north of Iran (Mazandaran) revealed midwives had more counseling and prescription practice on EC in comparison to doctors in the past 12 month. The most cited reason for prescription EC was condom rupture. Similarly, this study also showed midwives and family health workers more frequently counseled women about EC than GPs, as well as, screened women for unprotected sex. Also our study showed the most common cited reason for prescription of EC were rupture condom followed by none usage of contraceptive methods.

We found that GPs less attended on family planning training program than midwives and family health workers.

The GPs also less likely reported to screen women for unprotected sex and counseling about EC. The educational efforts should be conducted for GPs and more incorporate them in reproductive health programs.

Concerns regarding the repeated use of or dependence on EC Pills as a primary method of contraception have been raised. Study among Jamaican and Barbadian health staff showed about half who had ever refused to prescribe EC Pill, frequently cited reasons were medical contraindications to use, method unavailability, recent use, safety concerns and being uncomfortable prescribing it. Only one in five providers knew that the method could be safely used as often as needed and half of all providers believed that use of EC encourages sexual risk-taking and leads to increased sexually transmitted infection(STI) transmission (Yam et al., 2007). Similarly our study revealed majority of health staff worried the repeated use of EC pill would cause health risk.

The currently available evidence indicates that EC is safe and effective even when used several times (Shelton, 2002; Abuabara et al., 2004). The World Health Organization guidelines on EC services state that “repeated use of EC poses no health risks and should never be cited as a reason for denying women access to treatment” (WHO, 1998).

Repeat use may disclose the need for contraceptive counseling or for supplemental information about continuous methods (Abuabara et al., 2004). Getting a prescription for EC can be difficult and takes a long time. Giving EC to women in advance could guarantee that women have it on hand in case they need it (Polis et al., 2007) One opposition to making EC pills more widely available is the concern that women who know they can use EC pills may become reluctant with their regular contraceptive method. Reported evidence demonstrates convincingly that making EC pills more widely available does not increase risk taking behavior or adversely affect regular contraceptive use (Gold et al., 2004; Walsh & Frezieres, 2006).

Survey in Turkey indicated the few health staff included EC in routine consultations. Half of the health staff thought that people would not use effective contraceptive methods routinely and disseminating information about the EC would encourage young people to have unprotected sexual intercourse. Majority worried that increasing awareness of this method would lead to raising STI because people would stop using barrier methods (Sevil et al., 2006). The potential role of EC in preventing unintended pregnancy should be emphasized, particularly among countries like Iran with restricted abortion law. There is strong evidence that shows women's increased use of EC has substantially contributed to the 11% decline in abortion rates between 1994 and 2000 (Jones, 2002).

Our findings suggest the family planning training program should be conducted to all health staff, especially those are less knowledgeable. Educating health staff about modern contraceptive methods particularly EC may dispel some misconception about the methods, then they can convey accurate information to the clients and contribute women’s contraceptive decision making.
